# Transdural Lumbosacral Disc Herniation: When a Benign Pathology Raises Diagnostic and Therapeutic Challenges

**DOI:** 10.7759/cureus.84074

**Published:** 2025-05-14

**Authors:** Abdulrahman A Shamakh, Riadh M Rebai, Ahmed M Felemban, Ahmed T Khodri, Taghreed A Alsinani

**Affiliations:** 1 Medicine and Surgery, Ibn Sina National College for Medical Studies, Jeddah, SAU; 2 Department of Neurosurgery, King Fahad General Hospital, Second Health Cluster, Jeddah, SAU

**Keywords:** cauda equina syndrome, lumbosacral spine, radiculopathy, spinal cord compression, transdural disc herniation

## Abstract

Transdural disc herniation (TDH) is a very rare condition characterized by the displacement of intervertebral disc material transpiercing the thecal sac. A 33-year-old female patient presented to our emergency room with cauda equina syndrome. Upon imaging, we concluded that her clinical presentation was related to an L5-S1 ruptured disc herniation. Through an interspinous surgical approach, we identified a transdural disc herniation that was excised without postoperative complications. We reviewed the literature to highlight special imaging features that may aid in the diagnosis of this particular type of disc herniation and to plan a tailored surgical approach that may improve functional prognosis.

## Introduction

One of the scarce features of disc herniation is when the disc migrates through the dural sac. Transdural disc herniation (TDH) is an unusual pathology that accounts for only 0.3%-1.5% of cases, making the preoperative diagnosis and surgical planning more challenging [1,2]. Several factors contribute to the difficulty of obtaining a preoperative diagnosis: the nonspecific clinical presentation, ranging from the more common low back pain with sciatica to the more alarming cauda equina syndrome and the atypical imaging findings along with certain anatomical variations at the time of diagnosis [1,3]. Using a surgical approach for TDH that is similar to that for a common disc herniation may lead to significant intraoperative difficulties and complications. We present our case of TDH, discussing the clinical presentation and how we managed it despite lacking a preoperative diagnosis. We also discuss the outcome in the context of the limited literature available, highlighting differences from more common anatomical presentations of lumbar disc herniation. Throughout our case, we highlight a very rare feature of disc herniation that poses a diagnostic challenge in developing an effective surgical plan to achieve better results.

## Case presentation

A 33-year-old woman with no significant medical history presented to the emergency department with persistent low back pain and left S1 sciatica. Four months prior, she fell while descending the stairs. She reported initial low back pain that gradually radiated to the posterior aspect of her left thigh two weeks later. This pain was associated with numbness. She had been treated conservatively at another institution without improvement in her symptoms. Her condition worsened as she experienced urinary and fecal retention four days before admission.

Upon examination, her motor strength was intact, particularly in the lower limbs, as she was able to mobilize independently. However, she had diminished sensation in the pubic, perianal, and posterior left thigh regions. Lumbar spine syndrome was noted, and the straight leg raise test was positive at 30° on the left.

**Figure 1:**
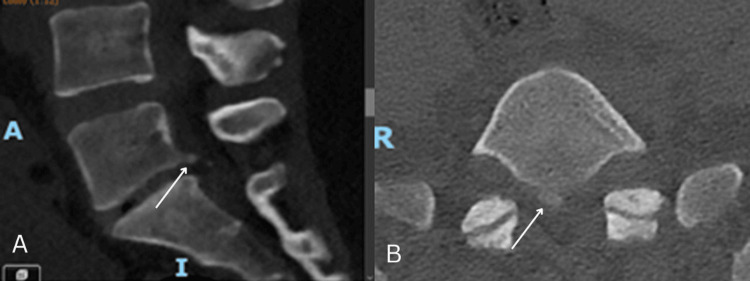
CT scan of the lumbar spine: (A) sagittal and (B) axial reconstructions. Detachment of the posterior and inferior margins of the L5 vertebra indicated by the arrow.

A computerized tomography of the lumbar spine scan showed an L5-S1 disc with reduced height and detachment of the posterior inferior margin of the L5 vertebra (Figure 1).

**Figure 2:**
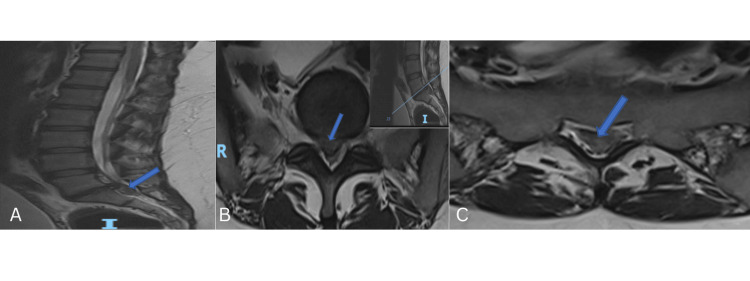
MRI of the lumbar spine. (A) Sagittal T2 sequence with blue arrow showing an isointense L5-S1 disc herniation with caudal migration. (B, C) Consecutive axial T2 sequences with blue arrows showing an isointense central disc extrusion filling the dural sac.

A prompt MRI of the lumbar spine revealed an isointense L5-S1 disc herniation with downward migration into the spinal canal on T2-weighted images (Figure 2).

Based on the clinical diagnosis of cauda equina syndrome related to an L5-S1 disc herniation, the patient was prepared for and underwent surgery within 24 hours.

**Figure 3:**
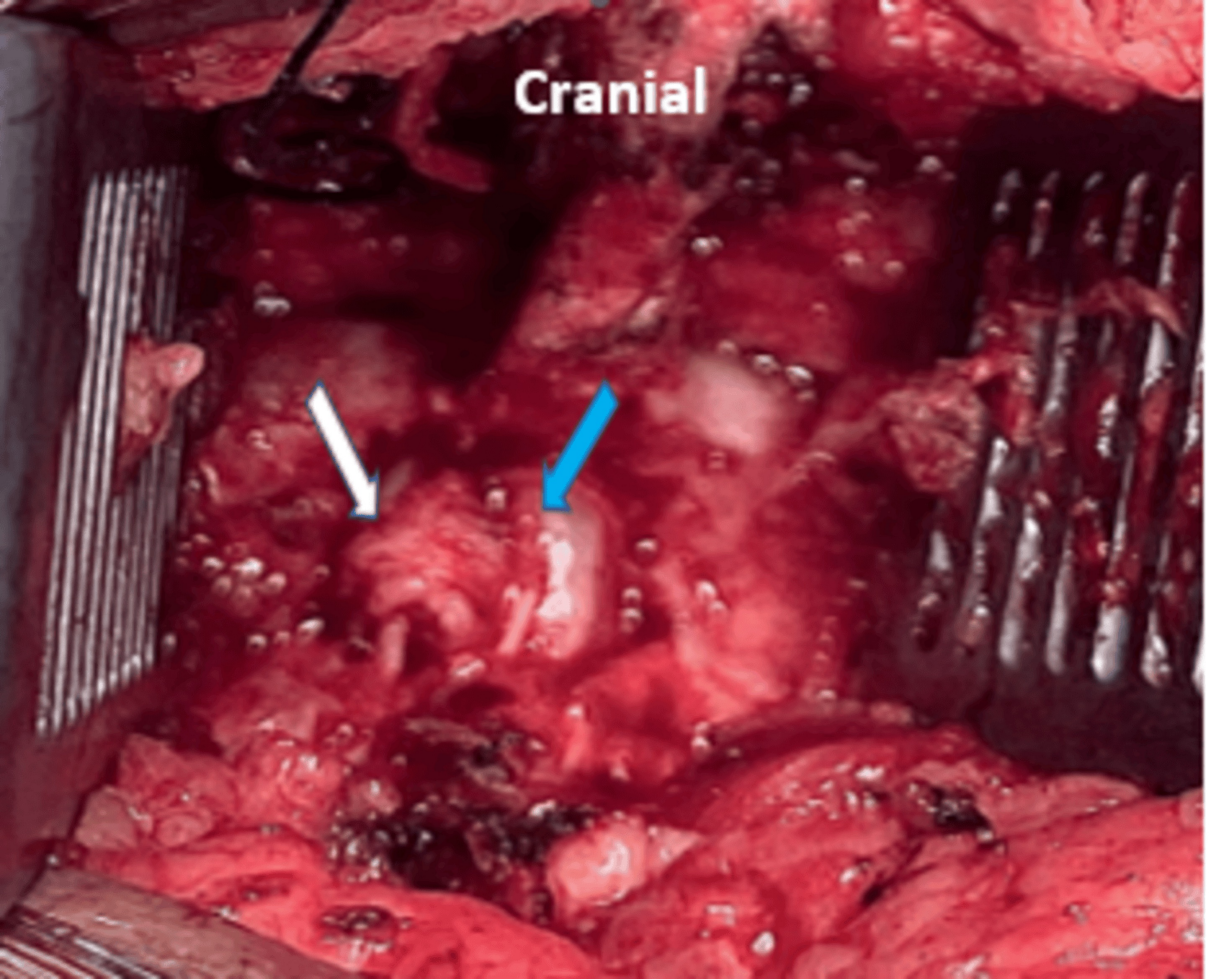
Intraoperative picture demonstrating a disc fragment (white arrow) penetrating the dura sac in its central aspect (blue arrow).

We planned an L5/S1 microdiscectomy via an interspinous process approach. We performed a partial laminectomy of L5 and S1 and excised the medial aspects of the L5-S1 facet joints bilaterally. Removal of the ligamentum flavum exposed the dura of the thecal sac, which was transpierced by the disc fragment protruding through its center (Figure 3). No adhesions were found intraoperatively. Although there was a dural defect, there was no evidence of cerebrospinal fluid (CSF) leakage.

**Figure 4:**
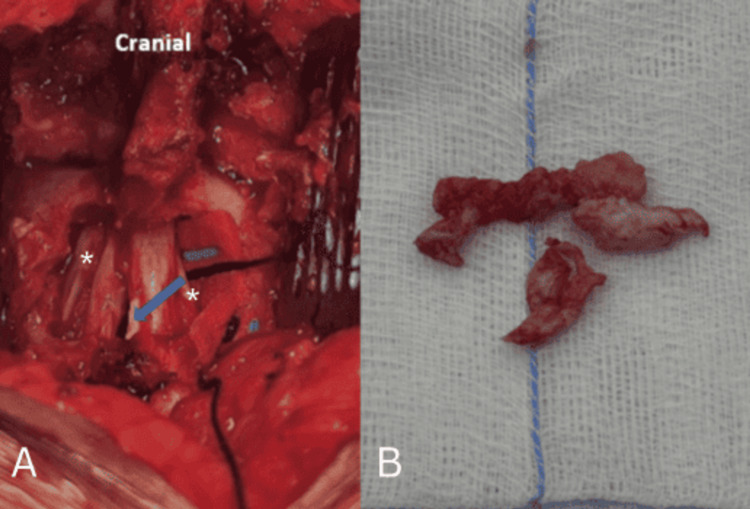
Intraoperative picture after removal of the disc fragments with the residual cavity in the central part of the thecal sac (blue arrow). The asterisks indicate S1 nerve roots.

The bulging disc fragment was meticulously dissected and removed (Figure 4) without CSF egress, as the path of the TDH was bordered by a capsule throughout the thecal sac. A layer of TachoSil® (Corza Medical, Parsippany, NJ, USA) was applied over the dural defect before standard closure.

Postoperatively, the patient was mobilized and demonstrated significant improvement in both low back and leg pain. Although the numbness in her leg lessened, it persisted in the perianal area. Due to urinary retention, bladder training was attempted but proved unsuccessful. The post-void residual (PVR) volume remained elevated at over 400 ml. Consequently, she was discharged home with an indwelling Foley catheter and scheduled for a follow-up appointment with a urologist. At the two-month postoperative follow-up visit to the urology clinic, the patient reported significant improvement in her urinary symptoms. The Foley catheter was successfully removed, and her PVR volume had decreased to 90 ml, indicating improved bladder function.

## Discussion

Transdural disc herniation is a documented phenomenon with over 150 cases reported since its initial description by Dandy in 1942 [3]. However, it remains a relatively rare complication compared to extradural disc herniation. TDH typically affects the lower lumbar spine, particularly between the fourth and fifth lumbar vertebrae (L4-L5) [4].

The exact etiology of TDH is unclear, but potential predisposing factors include dense adhesion between the dural sac and posterior longitudinal ligament, prior spinal surgery, congenital dural thinning, chronic disc herniation, and vertebral canal stenosis [4-7]. The absence of a definitive cause in some cases highlights ongoing research efforts to understand this phenomenon. Notably, our patient lacked a history of spinal interventions or known predisposing factors, although her symptoms had been progressing for four months following a lumbar trauma.

Intradural disc herniation (IDH) can present with similar symptoms to extradural disc herniation, but it carries a higher risk of cauda equina syndrome [8-10]. TDH can be further classified based on the extent of herniation as intra-arachnoid or extra-arachnoid. In the latter type, the disc material herniates through the dura but remains separated from the cerebrospinal fluid by the arachnoid membrane, often forming a capsule of inflammatory tissue. This extra-arachnoid TDH, as observed in our case, may not result in cerebrospinal fluid leakage during surgery for disc fragment removal [11].

Preoperative diagnosis of TDH remains challenging due to its ability to mimic intradural and extra-axial spinal lesions, such as schwannomas or metastases. Therefore, TDH should be included in the differential diagnosis for these masses [6]. MRI is the gold standard for diagnosis, with characteristic findings including the "hawk-beak" sign, crumb disc appearance, disruption of the posterior longitudinal ligament, and peripheral enhancement of the disc [1,12,13]. However, limitations in preoperative imaging can necessitate definitive diagnosis during surgery.

Surgical removal of the protruding disc fragment is the primary treatment for TDH. Conservative management may be considered in select cases as described by Borota et al. who reported a rare instance of an intradural herniated disc fragment seemingly resolving spontaneously [14]. Surgical challenges include dissection of the dural sac due to adhesions with the posterior longitudinal ligament and closure of the dural defect to avoid nerve injury [8-10,15]. In our case, these difficulties were not encountered because the specific type of TDH did not involve CSF egress. We managed the thecal sac defect with a layer of fibrin sealant patch (TachoSil®, Corza Medical).

The intraoperative management of a dural defect after excision of lumbar disc herniation varies depending on whether cerebrospinal fluid leakage is present or not. In cases with CSF egress, dural repair is required, typically using sutures, often reinforced with a sealant or glue, depending on the surgeon’s preference. Usage of fibrin glue in all cases of durotomy, irrespective of additional augmentation procedures like suturing, muscle patch, or gel foam, has become a standard practice. It has shown excellent results and has been demonstrated to be a safe and effective tool in various neurosurgical procedures. However, when no CSF leak is detected, as in our case where inflammatory granulation tissue spontaneously seals the defect around the posterior aspect of the dural sac, no further intervention is necessary.

In our case, the absence of a CSF leak, even with intraoperative Valsalva maneuvers, indicated that the natural sealing of the dural sac defect by the granulation tissue was stable. We opted to place a layer of TachoSil® over the defect, based on the rationale that it would prevent blood accumulation in the residual cavity and potentially avert a secondary CSF leak should the granulation tissue across the dural sac weaken.

Similar to common disc herniations, this subset of disc herniations can often go unsuspected preoperatively due to misleading and unusual imaging findings, leading to surgical challenges [1,2,4,15]. A limited exposure of the affected vertebral level may result in complications such as nerve injury, a prolonged surgery due to inadequate visualization for addressing the TDH and possible post-operative CSF leak or pseudomeningocele related to an inadequate repair of the dural defect through a tiny surgical approach.

Prognosis after TDH surgery varies depending on the type and duration of symptoms, as well as a history of prior spinal surgeries. Cases with cauda equina syndrome typically require longer recovery times, and surgical intervention should ideally occur within 48 hours of symptom onset to optimize outcomes [6,16]. Although she presented to our hospital a few days after the onset of sphincter disturbances, our patient experienced immediate and significant improvement in both lower back and leg pain postoperatively. Notably, some leg numbness persisted in the perianal area. Additionally, the patient reported a marked improvement in her urinary symptoms, allowing for the removal of the Foley catheter.

## Conclusions

Transdural disc herniation is a rare complication of lumbar degenerative disc disease. The presence of cauda equina syndrome, often associated with TDH, necessitates prompt surgical intervention. A preoperative diagnosis of TDH allows for a tailored surgical plan and minimizes the risk of complications. While diagnosis relies on identifying unusual features on spinal MRI, including TDH in the differential diagnosis of intra- and extradural lesions at the affected level is crucial. The prognosis for TDH can differ from that of common disc herniations, particularly regarding recovery time.
